# Sex differences in macrovascular responsiveness: Acute effects of hot water immersion versus aerobic exercise in overweight older adults

**DOI:** 10.1113/EP093959

**Published:** 2026-09-25

**Authors:** Juliene G. Costa, Joao Carlos Locatelli, Kristanti W. Wigati, Louise H. Naylor, Andrew Haynes, Helen Jones, Daniel J. Green

**Affiliations:** ^1^ School of Human Sciences The University of Western Australia Crawley Western Australia Australia; ^2^ Medical Physiology and Biochemistry Department, Faculty of Medicine Universitas Airlangga Surabaya Indonesia; ^3^ Research Institute for Sports and Exercise Science Liverpool John Moores University Liverpool UK

**Keywords:** acute exercise, heat therapy, overweight, passive heat, vascular physiology

## Abstract

Recent studies have focused on the potential benefits of heat exposure for cardiovascular health. However, the acute effects of heat therapy on conduit arteries remain unclear, particularly in overweight and obese older males and females. This study aimed to compare core temperature (*T*_c_), heart rate (HR), and brachial, femoral, and carotid artery diameter and velocity (Doppler ultrasound) responses to an acute exercise (EXE) session versus hot water immersion (HWI), and to determine whether physiological responses are sex‐dependent. Twenty‐five overweight older adults (10♂/15♀; age: 59 ± 7, BMI: 29.4 ± 3.4 kg/m^2^) underwent two experimental sessions (30 min): EXE (60–70% HR_max_) and HWI (40°C). HWI resulted in a larger increase in *T*_c_ across the session with the largest difference at 30 min (0.9 ± 0.3 vs. 0.7 ± 0.3°C; *P* = 0.024). No differences were observed in *T*_c_ between males and females (interaction *P* = 0.271). HWI induced a lower rise in HR (♂ 40 ± 18 vs. ♀ 51 ± 12 bpm at 30 min; *P* = 0.013) than EXE, and females exhibited a larger change in HR after HWI than males (♂ 27 ± 12 vs. ♀ 48 ± 17 bpm; *P* = 0.003). HWI also induced a larger reduction in systolic (−6 ± 10 vs. 1 ± 7 mmHg; *P* = 0.012) and diastolic BP (−10 ± 9 vs. −2 ± 5 mmHg; *P* = 0.001) than EXE, but with no sex differences. HWI induced a larger increase in brachial diameter, brachial and femoral velocity, brachial and femoral flow, and shear rate than EXE. Females exhibited significantly higher change in femoral velocity (♀ 26 ± 8 vs. ♂ 17 ± 6 cm/s; *P* = 0.011) and shear rate (♀ 157 ± 55 vs. ♂ 98 ± 38 cm/s; *P* = 0.008) in response to HWI than males. A single session of HWI induced larger vascular responses than exercise. Males and females both benefit, but somewhat differently and possibly via different mechanisms.

## Abstract (highlights)

- **What is the central question of this study?**
  What are the acute effects of heat therapy on conduit arteries, particularly in overweight and obese older males and females?
- **What is the main finding and its importance?**
  A 30 min‐session of hot water immersion induced greater vascular responses than cycling exercise and revealed sex‐related differences. Females exhibited greater femoral velocity and shear rate despite a similar increase in core temperature. Whole‐body heating may provide a lower‐heart‐rate alternative to aerobic exercise for enhancing arterial shear stress in older overweight adults, with potential diferences between males and females.

## 1 INTRODUCTION

Exercise training is well established as a physiological stimulus to improve cardiovascular health and reduce the long‐term risk of cardiovascular disease (Tu et al., 2025). One proposed mechanism explaining this relationship involves the improvement in endothelial function in both active and inactive regions, primarily through an increase in shear stress, specifically, an increase in forward directional (antegrade) shear stress (Green et al., 2017). We previously demonstrated that increases in shear rate, regardless of the type of exercise, improve conduit artery function in healthy participants (Thijssen et al., 2009). Additionally, the type of exercise can influence the magnitude and patterns of shear and flow (Thijssen et al., 2009). While the cardiovascular benefits associated with physical activity are well established, some individuals with medical conditions may have difficulty exercising and obtaining cardiovascular benefits due to the inability to engage in or perform activity at an intensity high enough to induce cardioprotective effects (Wen et al., 2025). This is particularly relevant for individuals with obesity, who face heightened risk for cardiovascular disease, especially as they age (Tu et al., 2025). Understanding how different modalities, including exercise and heat exposure, compare in their effects on cardiovascular health for this population is crucial for developing effective interventions tailored to their unique needs and limitations.

In a series of studies, our group has previously established that the acute effects of heat stress can lead to several significant physiological responses in humans, including increased core temperature (Carter et al., 2014a), cutaneous blood flow (Green et al., 2010) and arterial shear rate (Carter et al., 2014a; Naylor et al., 2011), to levels comparable to those achieved during an acute exercise bout (Carter et al., 2013; Tinken et al., 2009). These studies have primarily focused on healthy young males and have assessed brachial artery responses; there is less data on vascular responses in the lower limbs (Kröger et al., 1999). It is currently unknown whether haemodynamic responses to heat stress vary across vascular beds within the same individuals.

Recent investigations have also highlighted the possibility that vasodilator responses to acute heating may be age‐ and sex‐dependent. Koch Esteves et al. (2023) compared trained elderly (69 ± 5 years) participants to a young (26 ± 7 years) comparison group and reported that age‐related structural and functional alterations were evident in conduit arteries. In another study Atkins et al. (2024) compared the impact of age and sex on responses to heat stress (3 h at 47°C, 15% relative humidity; replicating the heat waves in Los Angeles in 2018) and found that older females exhibited a greater increase in heart rate to heat stress. In contrast, young females exhibited greater sweat loss than their male counterparts in response to peak heating conditions (47°C, 15% relative humidity) during daily activities (3 h of low‐intensity walking). These data suggest that physiological differences may exist in the response to exercise and heat exposure between men and women. However, it was recently reported that only ∼15% of studies on human thermoregulation have recruited women (Hutchins et al., 2021), even though older women who are overweight exhibit a high prevalence of chronic disease (Wilson et al., 2002). Hence, there is a need to understand the differences and similarities in the vascular responsiveness in males and females in response to exercise and heat exposure, as well as the potential protective vascular effects of these protocols for this population.

The aim of the present study was to investigate the acute effects of 30 min of heat versus the same period of exercise in brachial, femoral and carotid haemodynamics alongside core temperature, heart rate and blood pressure of aged, overweight males and females. We hypothesised that the exercise session would elicit more pronounced responses than the hot water immersion (HWI) in terms of vascular diameter, flow and shear rate. In addition, despite the same environmental exposure, males would exhibit lower core temperature (*T*_c_) and reduced vascular responses than females.

## 2 METHODS

### 2.1 Participants

Twenty‐five (10 males, 15 post‐menopausal females) overweight and obese participants aged 45–70 years were recruited from the local community through advertising. Inclusion criteria were: individuals with cardiovascular risk factors (Cosentino et al., 2023; Kivimäki et al., 2017) of a body mass index (BMI) ≥27 kg/m^2^ with ≥1 weight‐related comorbidity (e.g., hypertension, dyslipidaemia or obstructive sleep apnoea), post‐menopausal women (more than 12 months without menstruation), non‐smokers, relatively physically inactive (<150 min per week of moderate intensity exercise) and no chronic experience with heat therapy in the last 6 months. Exclusion criteria were: type 2 diabetes, use of menopause hormone therapy, or a cardiovascular condition that prevents the participant from participating in exercise interventions, such as uncontrolled hypertension, recent myocardial infarction, or unstable angina. In keeping with recent recommendations (Hagstrom et al., 2025), individuals reported their gender, which was consistent in all cases in this study with their sex recorded at birth.

After participants contacted the research team to express their interest in joining the study and were confirmed eligible, they were invited for an initial in‐person meeting at the university. During this session, we offered a thorough explanation of the study to ensure clarity, and written informed consent was obtained. A pathology form was provided to each participant, instructing them to attend a specific pathology laboratory (off‐site) in the morning following an overnight fast within the next 7 days. This facilitated the testing of fasting blood glucose, glycated haemoglobin (HbA1C) and lipid levels. The blood test was used to characterise the sample and confirm the inclusion criteria. This study conformed to the standards outlined in the *Declaration of Helsinki* and was reviewed and approved by The University of Western Australia Human Research Ethics Committee (2022/ET000738).

### 2.2 Experimental protocol

**FIGURE 1:**
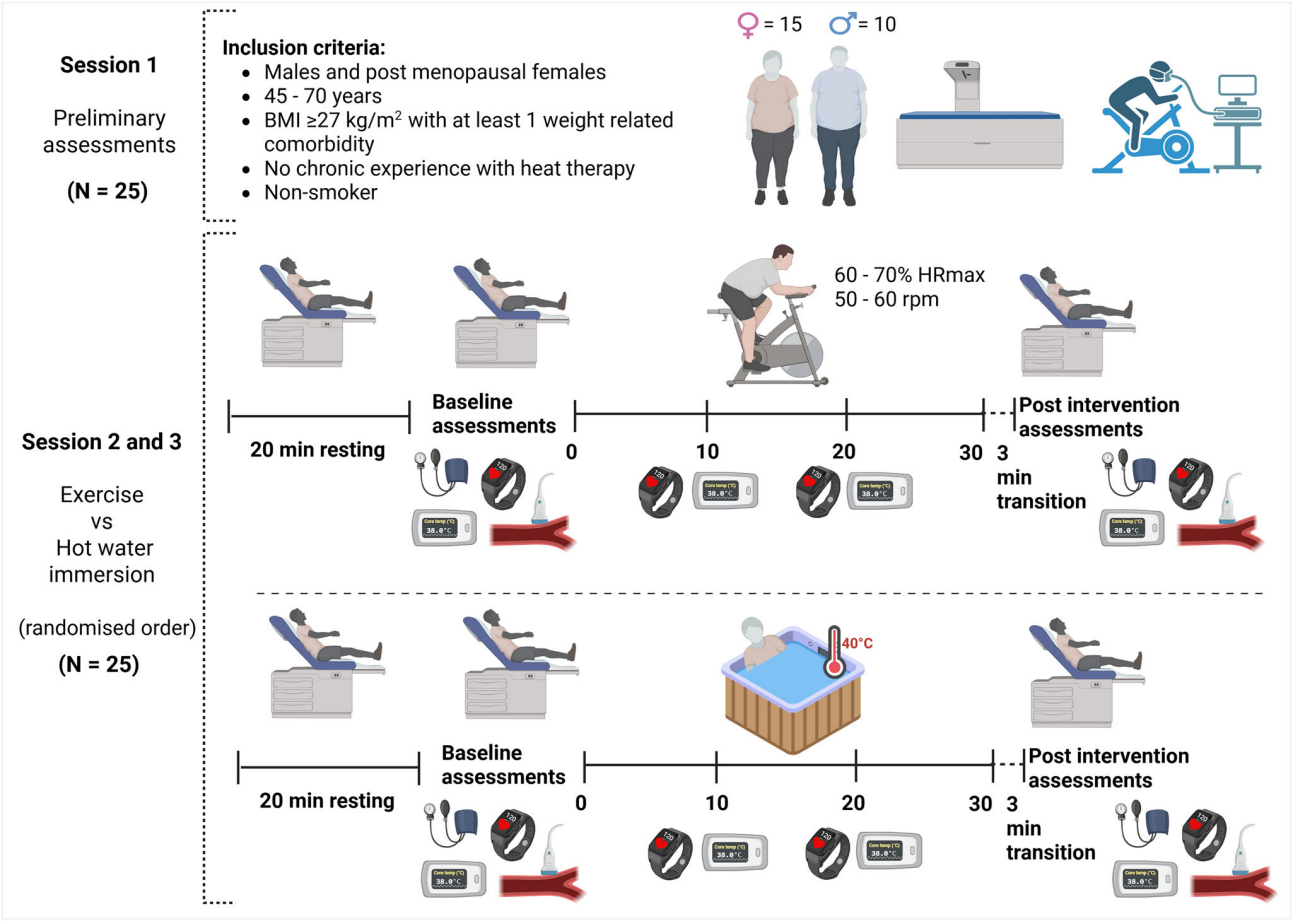
Study design.

This randomised crossover study involved two experimental sessions: HWI and exercise (EXE). Each participant attended three laboratory sessions (Figure 1) at the Cardiovascular Research Laboratory at The University of Western Australia.

### 2.3 Preliminary assessments

Preliminary assessments were conducted 7 days before the experimental session in a thermoneutral laboratory at The University of Western Australia. This session involved measuring height and body mass (used to calculate body mass index as kg/m^2^), body composition by dual‐energy X‐ray absorptiometry (DXA), and aerobic capacity. Body composition was assessed using (Lunar iDXA, GE Healthcare, Madison, WI, USA), which derived indices including fat mass, lean mass and visceral adipose tissue. After participants completed a DXA scan, they were instrumented before having their aerobic capacity assessed ($\dot{V}O_{2}\max$). $\dot{V}O_{2}\max$ was assessed during a continuous incremental cycle test on an upright cycle ergometer (Corival ergometer, Lode, Groningen, Netherlands). The test required participants to exercise at gradually increasing workloads, starting with 50 W at 60 rpm, and increasing by 25 W every 2 min until volitional exhaustion was reached. Heart rate (HR) was measured using a Polar H10 (Polar Electro Oy, Kempele, Finland) and respiratory flow and gas composition (Parvomedics TrueOne 2400, Salt Lake City, UT, USA) were measured during expiration throughout the test, with HR and rating of perceived exertion (RPE, 6–20 Borg scale) being recorded in the last 15 s of each stage. The maximum HR achieved in the $\dot{V}O_{2}\max$ test was used to prescribe the exercise session, being individualised for each participant.

### 2.4 Experimental sessions

All participants attended two experimental sessions in random order, which took place at the same time each day, with least 72 h between sessions to reduce any residual effects from the previous session. The experimental sessions were matched in stimulus time (30 min) and included either a HWI or EXE session. The HWI session consisted of sitting with legs straight for 30 min in a water bath (San Francisco Smart Luxe HydroJet Pro 7, Bestway Inflatables & Material Corp., Shanghai, China) set at a temperature of 40°C up to the level of the xiphoid process, with both arms submerged. The EXE session consisted of 30 min of upright leg cycling at 50–60 rpm at an intensity of 60–70% of the maximum HR obtained from the $\dot{V}O_{2}\max$ test. The targeted heart rate zone was reached with a progressive increase in intensity (in watts) in the initial 2 min of the protocol and adjusted as needed according to HR, with a cadence of approximately 60 rpm. No fan or air conditioning was used during the sessions.

Before each experimental session participants were instructed to take their regular daily medications at the same time of the day preceding the experiments, and were advised to avoid caffeine and strenuous physical activity for 24 h, and to refrain from eating for 3 h prior to the session. Participants were also instructed to wear shorts and, if comfortable, be shirtless, and females were asked to wear a sports bra and use the same outfit during both sessions. To measure core temperature (*T*_c_) a temperature sensor telemetry capsule (eCelsius Performance electronic capsule; BodyCap Medical, Hérouville‐Saint‐Calir, France) was delivered to each participant on the night before the test and instructed to ingest it approximately 6–7 h before the experimental session. This ingestion time was monitored and confirmed, to ensure that the sensor was at an appropriate location in the digestive tract during data collection (Byrne & Lim, 2007).

Baseline and post‐session assessments were conducted with participants lying in a semi‐reclined position outside the bath and away from the bike. Participants completed a 20‐min rest before baseline measures. After the baseline measures, participants engaged in 30 min of intervention. On completion, they had 3 min to transition to the post‐intervention assessments (get out of the bath or bike, quickly dry off and lie down). The transition durations were similar between trials.

Baseline and post‐intervention assessments included: systolic (SBP) and diastolic (DBP) blood pressure (Dinamap V100, GE Healthcare), *T*_c_ and heart rate (HR; Polar H10 HR monitor, Polar Electro Oy). HR and *T*_c_ readings were taken at 10‐min intervals during both the HWI and EXE sessions (i.e., 10, 20, and 30 min). During the HWI session, participants assessed their thermal comfort (ThC) and thermal sensation (ThS) using perceptual scales (Epstein & Moran, 2006), rating overall feeling from −5 (very bad) to +5 (very good) and comfort level with temperature on a scale from −4 (very uncomfortable, cold) to +4 (very uncomfortable, hot), with 0 being neutral (comfortable). During the EXE session, the Borg 6–20 RPE scale was used to monitor the session intensity (Shigematsu et al., 2004).

#### 2.4.1 Arterial high‐resolution ultrasound scanning

The diameter and velocity of the common carotid (CCA) (proximal to the bifurcation), brachial (BRA) and femoral (FEM) arteries was assessed on the right side following recommended guidelines (Thijssen et al., 2011, 2019). The BRA and FEM arteries were scanned simultaneously by two sonographers using two ultrasound machines, followed by scanning of the CCA (uSmart3300, Terason, Burlington, MA, USA). The 1‐min scan was performed using a high‐resolution 15 MHz linear‐array transducer at an insonation angle of 60°. The site scanned at baseline was marked and maintained for subsequent scans to ensure the same portion of each artery was used in the analysis. Screen capture software (Camtasia Studio; TechSmith, Okemos, MI, USA) recorded a continuous video file for later analysis using a custom‐designed edge‐detection and wall‐tracking software package to calculate the blood flow in the BRA, FEM and CCA arteries (Woodman et al., 2001). Blood flow was calculated from synchronised diameter and velocity data using the product of lumen cross‐sectional area and Doppler velocity.

### 2.5 Data analysis

The sample size calculation was based on data from the study by Ayme et al. (2014), which reported an increase in BRA artery diameter after HWI at the level of the xiphoid (mean ± SD: 4.0  ±  0.2 vs. 3.7  ±  0.2 mm; effect size *f* = 0.75). Based on these data, a minimum of 20 participants in total (considering calculation for two groups and two measures) was required to reject the null hypothesis at 95% power and α = 0.05. G*Power software (version 3.1.9.7) was used for calculating the sample size. Analysis of the Studentized residuals showed normality, as assessed by the Shapiro–Wilk test, and no outliers, as assessed by no Studentized residuals exceeding ±3 standard deviations. There was sphericity for the interaction term, as assessed by Mauchly's test of sphericity (*P *< 0.05). Data were expressed as means ± standard deviation. Student's unpaired *t*‐test was used to compare differences between females and males in baseline characteristics and [paired t‐tests were used] to compare baseline measurements across experimental sessions. A two‐way repeated‐measures ANOVA was performed to compare differences between HWI and EXE at baseline and after the sessions for HR, *T*_c_, and the BRA, FEM and CCA arteries. A two‐way mixed ANOVA was used to compare changes (deltas) in males and females across HWI and EXE sessions. Person's correlation was conducted to examine the relationship between the changes (Δ) in *T*_c_ with BRA and FEM velocity, flow, shear rate and antegrade shear. For all comparisons, significance was set at *P *< 0.05. Statistical analyses were performed using SPSS Statistics version 29.0 (IBM Corp., Armonk, NY, USA).

## 3 RESULTS

### 3.1 Participants characteristics

A total of 25 participants (10♂ and 15♀; age 59 ± 7; BMI of 29.4 ± 3.4 kg/m^2^) were recruited and completed the two experimental sessions; baseline characteristics are presented in Table 1. The males were heavier and taller, with greater body surface area, greater lean mass (total, arms and legs), and less fat mass on the arms and legs. The total fat mass and BMI were similar in males and females. The males were fitter, as indicated by both absolute and relative $\dot{V}O_{2}\max$. The baseline measurements for females and males in both experimental sessions are presented in Table S1. Females exhibited significantly lower DBP, smaller baseline BRA diameter, velocity and shear rate and smaller FEM diameter before the exercise session compared to males. At baseline, before the HWI session, females exhibited smaller BRA diameter, shear rate and CCA diameter compared to males. All participants completed both experimental sessions without adverse events.

**TABLE 1:** Participant characteristics (*n* = 25).

	All participants (*n* = 25)	Females (*n* = 15)	Males (*n* = 10)	F versus M *P*‐value
Age (years)	59 ± 7	58 ± 7	60 ± 8	0.615
Weight (kg)	84.4 ± 11.5	80.1 ± 9 4	91.5 ± 11.6	**0.015**
Height (m)	1.69 ± 0.08	1.65 ± 0.04	1.77 ± 0.07	**<0.001**
BMI (kg/m^2^)	29.4 ± 3.4	29.4 ± 3.6	29.3 ± 3.1	0.967
BSA (m^2^)	1.9 ± 0.2	1.9 ± 0.1	2.1 ± 0.2	**<0.001**
Total lean mass (kg)	47.8 ± 7.3	43.4 ± 3.0	55.1 ± 6.4	**<0.001**
Arms lean mass (kg)	5.2 ± 2.0	4.5 ± 0.4	6.2 ± 1.5	**<0.001**
Leg lean mass (kg)	16.4 ± 6.5	14.9 ± 1.5	18.9 ± 2.8	**<0.001**
Total fat mass (Kg)	33.1 ± 8.3	33.4 ± 7.9	32.8 ± 9.3	0.868
Arms fat mass (kg)	3.3 ± 1.0	3.6 ± 0.8	2.9 ± 0.6	**0.043**
Leg fat mass (kg)	9.4 ± 2.5	10.4 ± 2.5	7.9 ± 2.1	**0.020**
$\dot{V}O_{2}\max$ (L/min)	2.3 ± 0.8	1.9 ± 0.5	2.9 ± 0.7	**0.001**
$\dot{V}O_{2}\max$ (ml/kg/min)	27.1 ± 8.3	24.0 ± 7.2	31.7 ± 7.9	**0.019**
Cholesterol (mmol/L)	5.3 ± 0.9	5.6 ± 0.7	5.0 ± 1.0	0.135
Triglycerides (mmol/L)	1.2 ± 0.5	1.2 ± 0.5	1.3 ± 0.6	0.782
HDL (mmol/L)	1.6 ± 0.4	1.8 ± 0.3	1.4 ± 0.3	**0.014**
LDL (mmol/L)	3.2 ± 0.8	3.3 ± 0.8	3.0 ± 0.7	0.474
Glucose (mmol/L)	5.1 ± 0.5	5.1 ± 0.6	5.1 ± 0.4	0.879
HbA1c (%)	5.3 ± 0.3	5.4 ± 0.4	5.3 ± 0.2	0.685
Medical conditions (taking medication)				
Hypothyroidism	5 (5)	5 (5)	0	
Hypertension	6 (5)	3 (3)	3 (2)	
High cholesterol	14 (4)	8 (2)	6 (2)	
Reflux	3 (3)	1 (1)	2 (2)	
Articular diseases	6 (0)	5 (0)	1 (0)	
Mental health diseases	2 (2)	2 (2)	0	
Asthma	2 (1)	1 (0)	1 (1)	

*Note*: Values are presented as means ± standard deviation. Unpaired *t*‐test; significance was set at *P *< 0.05.

Abbreviations: BMI, body mass index; BP, blood pressure; BSA, body surface area; HbA1c, glycated haemoglobin; HDL, high‐density lipoprotein; LDL, low‐density lipoprotein.

### 3.2 Differences between EXE and HWI

**FIGURE 2:**
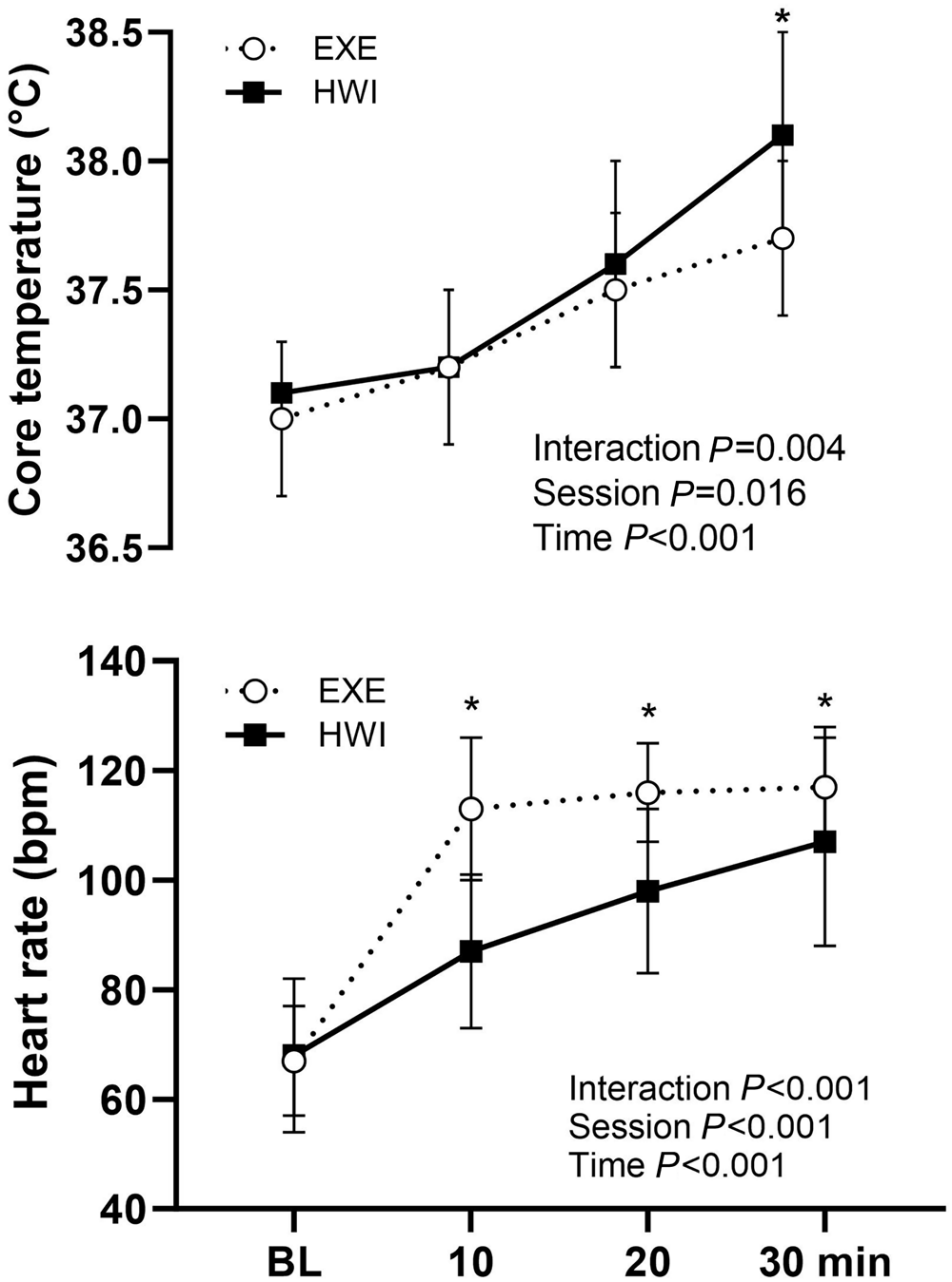
Core temperature (*T*_c_) and heart rate (HR) in response to exercise (EXE) and hot water immersion (HWI). ANOVA two‐way; *post‐hoc significance of *P *< 0.05. Data acquired from 25 participants.

Participants performed the experimental sessions under similar room‐temperature conditions (EXE: 23.4 ± 1.6 vs. HWI: 22.3 ± 4°C, *P* = 0.197) and relative humidity (EXE: 50 ± 6 vs. HWI: 52 ± 13%, *P* = 0.485). HWI increased *T*_c_ more than EXE, exhibiting significant session (*P* = 0.016), time (*P *< 0.001) and interaction (*P* = 0.004) effects (Figure 2). *Post hoc* analysis revealed a significant difference in *T*_c_ between EXE and HWI at 30 min (ΔHWI: 0.9 ± 0.3°C vs. ΔEXE 0.7 ± 0.3°C, *P* = 0.024), with no significant differences at baseline, 10 or 20 min. There were significant effects on HR for session (*P *< 0.001), time (*P *< 0.001) and interaction (*P *< 0.001) (Figure 2). Both sessions resulted in significant increases in HR from baseline, but the EXE session exhibited significantly higher values than HWI at all time points (i.e., 10, 20 and 30 min), across the 30‐min session, with a rise at the end of the session in ΔEXE: 51 ± 12 vs. ΔHWI: 40 ± 18 bpm (*P* = 0.013). Both sessions reduced systolic (Δ −6 ± 10 vs. ΔEXE: 1 ± 7 mmHg, *P* = 0.012) and diastolic blood pressure (Δ −10 ± 9 vs. ΔEXE: −2 ± 5 mmHg, *P* = 0.001) at 30 min.

**FIGURE 3:**
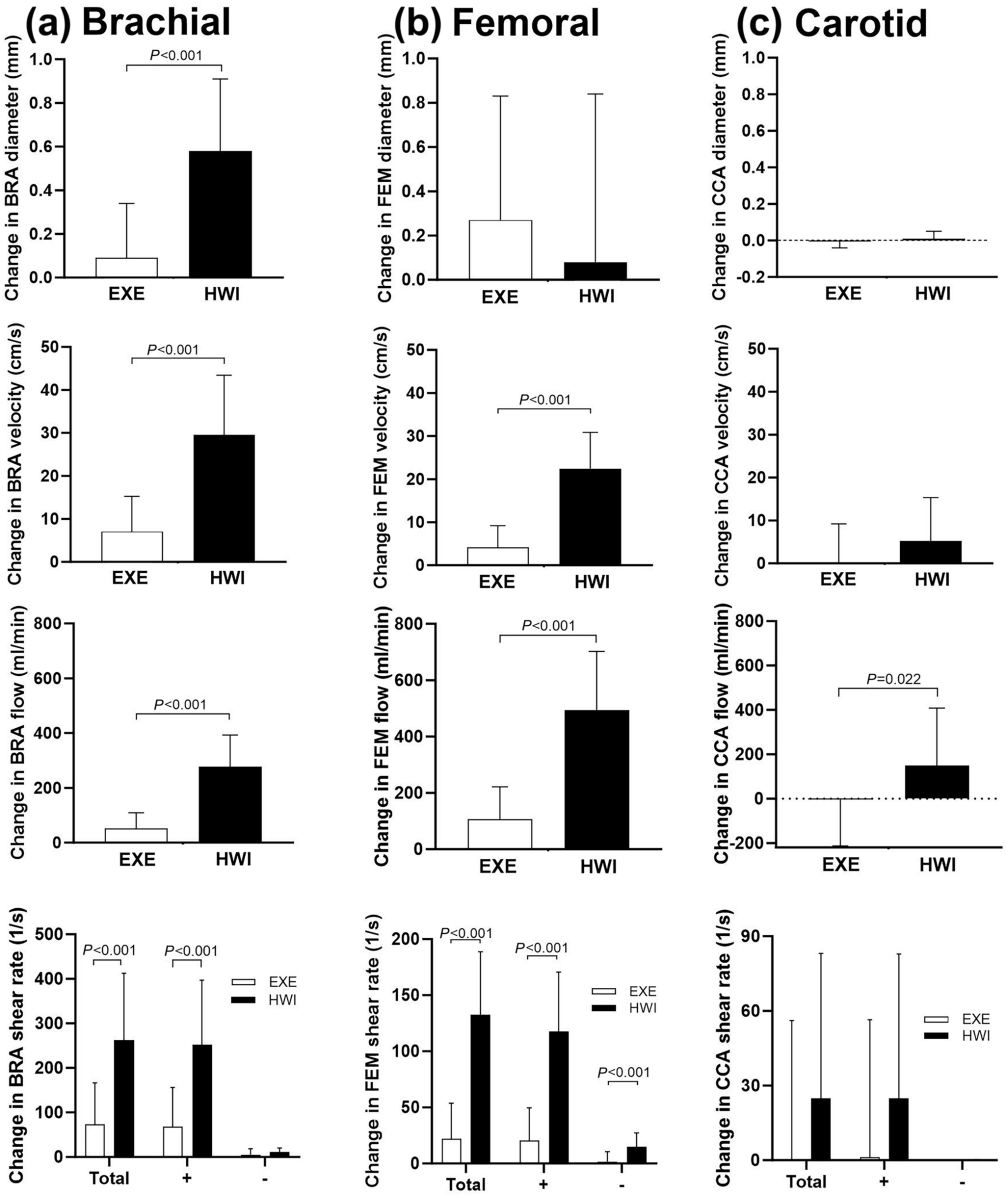
Difference between exercise (EXE) and hot water immersion (HWI) sessions in changes in diameter, velocity and flow and shear rate of the brachial (BRA), femoral (FEM) and common carotid (CCA) arteries. +, antegrade shear; −, retrograde shear. *P*‐values obtained by paired *t*‐test.

HWI resulted in a larger increase in BRA artery diameter (*P *< 0.001), blood velocity (*P *< 0.001), blood flow (*P *< 0.001) and shear rate (*P *< 0.001) than the EXE session (Figure 3a). The changes in shear rate were due to an increase in antegrade shear (ΔHWI: 252 ± 145 vs. ΔEXE: 68 ± 88 s^−1^, *P *< 0.001), and no difference in retrograde shear (ΔHWI: 11 ± 10 vs. ΔEXE: 5.3 ± 13, *P* = 0.114) (Figure 3a). No significant difference was observed in FEM diameter in response to either session; however, HWI induced a larger change in FEM velocity (*P *< 0.001), flow (*P *< 0.001) and shear rate (Δ 133 ± 56 vs. 22 ± 32 s^−1^, *P *< 0.001), antegrade (Δ 118 ± 53 vs. 20 ± 29 s^−1^, *P *< 0.001) and retrograde shear (ΔHWI: −15 ± 12 vs. ΔEXE: −2 ± 9 s^−1^, *P *< 0.001) than EXE (Figure 3b). The HWI session also resulted in a larger increase in CCA flow than EXE (Δ 150 ± 259 vs. −3.0 ± 210 mL/min, *P* = 0.022) (Figure 3c). There was no significant difference between sessions in CCA diameter (*P* = 0.155), velocity (*P* = 0.051) and shear rate (*P* = 0.102). Correlation analysis indicated a positive, moderate relationship between Δ*T*_c_ and BRA ΔShear rate (1/s) *r*(22) = 0.417, *P* = 0.043 in response to EXE session. However, this association was not found for BRA shear rate, velocity, flow or antegrade shear in response to HWI session. Additionally, no associations were observed in FEM parameters in response to either the EXE or HWI sessions.

### 3.3 Sex differences in response to EXE and HWI

Males and females increased *T*_c_ similarly (group *P* = 0.140) in response to both sessions (session *P* = 0.055), with no interaction effect (*P* = 0.271). The increases in HR were similar between sexes in response to EXE; however, in response to HWI, males exhibited a lower increase in HR compared to females (Δ♂ 27 ± 12 vs. Δ♀: 48 ± 17 bpm; *P* = 0.003). Males and females had similar increases in thermal sensation, thermal comfort, RPE, and systolic and diastolic blood pressure.

**FIGURE 4:**
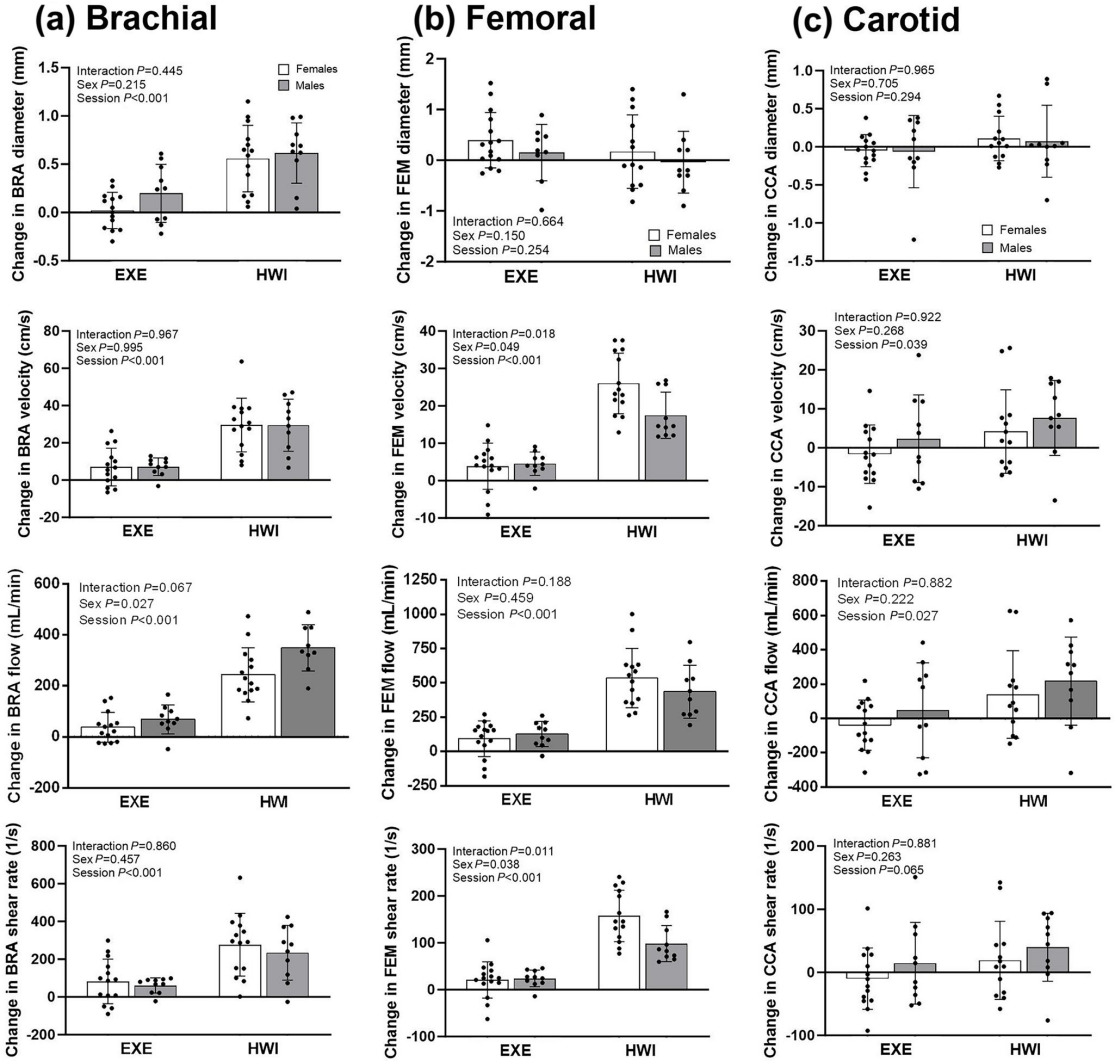
The difference between females and males in response to exercise (EXE) and hot water immersion (HWI) on the diameter, velocity and flow and shear rate of the brachial (BRA), femoral (FEM) and common carotid arteries (CCA). Data acquired from 15♀ and 10**♂**.

No sex differences (Figure 4) were exhibited in BRA diameter (interaction *P* = 0.445), velocity (interaction *P* = 0.967), flow (interaction *P* = 0.067) or shear rate (interaction *P* = 0.860). In the FEM artery, no sex differences (sex *P* = 0.150) were exhibited in diameter (interaction *P* = 0.664). However, for FEM velocity, an interaction (*P* = 0.018), sex (*P* = 0.049) and session (*P *< 0.001) effect showed greater change in velocity after HWI in females than males (Δ♀ 26 ± 8 vs. Δ♂ 17 ± 6 cm/s, *P* = 0.011) (Figure 4a). In addition, for FEM artery shear rate, an interaction (*P* = 0.011), sex (*P* = 0.038) and session (*P *< 0.001) demonstrated that females had a larger magnitude of response than males (Δ♀ 157 ± 55 vs. Δ♂ 98 ± 38 s^−1^, *P* = 0.008). In CCA parameters, no sex differences were exhibited in response to HWI and EXE (Figure 4c).

## 4 DISCUSSION

We compared the acute effects of a bout of 30 min of whole‐body heating via HWI to aerobic EXE on an upright leg cycle ergometer in older, overweight males and females. Our main findings were the following. (1) After 30 min of exposure, the HWI session induced greater impacts than EXE on BRA artery diameter, flow and shear rate, and in FEM artery flow and shear rate. Additionally, we observed a larger increase in *T*_c_ and reduction in systolic and diastolic blood pressure after HWI, and higher HR after EXE. (2) In response to HWI, males exhibited a lower increase in HR compared to females.

We compared conditions that were ecologically valid, in that they are typical of the type of activities undertaken by older overweight and obese individuals for desired health benefits. Whilst this meant that they were not strictly physiologically matched, the *T*_c_ response we observed was similar during the sessions, with differences only emerging in the last 10 min and the average difference between conditions in Δ*T*_c_ at 30 min being ∼0.2°C. HWI resulted in larger increases in BRA and FEM parameters than EXE, while HR was higher in the EXE session. These findings are consistent with the study by Thomas et al. (2016) in which young lean participants (27 ± 5 years; 24.4 ± 1.5 kg/m^2^; *n* = 10) underwent 30‐min sessions of HWI at waist level (42°C) and treadmill exercise (65–75% age‐predicted HR_max_). The BRA artery was not analysed in that study. The study revealed that the FEM shear rate increased more significantly with HWI (+181 ± 23 vs. +104 ± 23 s^−1^), and the authors concluded that this was attributed to the HWI treatment, which increased *T*_c_ and muscle temperatures beyond the levels achieved with EXE alone. Chiesa et al. (2016) have suggested that increased leg blood flow during heating is not only associated with higher *T*_c_ and muscle temperature, but also that much of the leg blood flow increase is activated by local mechanisms after direct heating at the measurement site; increases in flow occur prior to notable *T*_c_ elevation and centrally mediated reflex responses. These findings support the importance of local temperature effects on arterial haemodynamic responses to heating (Menzies et al., 2025) and clarify why femoral and brachial arm parameters in our study were significantly higher after the HWI session than after EXE. This increase is attributed to the combined effects of elevated stimulus for the rise in *T*_c_, and local heating from water in contact with the skin, which together trigger local and central mechanisms for blood flow regulation and heat dissipation. These physiological responses that promote vascular health are relevant in contexts where interventions such as EXE may be difficult to perform or contraindicated (Wen et al., 2025).

In the current study, the significant increases we observed in shear rate through both BRA and FEM arteries in response to HWI were due to an increase in antegrade shear compared to the EXE session. Francisco et al. (2021) also demonstrated large increases in antegrade shear in conjunction with marked reductions in retrograde shear during HWI, versus robust increases in antegrade shear and minor increases in retrograde shear during EXE. Another study reported similar increases in FEM blood flow and shear response profiles when comparing HWI to semi‐recumbent stepping (Amin et al., 2021) and treadmill EXE (Thomas et al., 2016). Studies have differed in methodology; for example, we did not measure blood flows immediately after the sessions, taking 3 min to allow participants to transition to the post‐intervention assessments, from the bath or bike, quickly dry off and lie down, which could underestimate the EXE effects. According to Amin et al. (2021) there is a rapid reduction in blood flow following cessation of muscle contraction.

In our study, the difference in core temperature change (Δ*T*_c_) between conditions was significant, although not substantial (0.9 ± 0.3 vs. 0.7 ± 0.3°C, *P* = 0.024). Conversely, HR increased much more after the EXE session than after the HWI (EXE: 51 ± 12 vs. HWI: 40 ± 18; *P* = 0.018). These findings highlight the distinct physiological responses elicited by each stimulus. A recent study (Amin et al., 2022) with healthy moderately trained adults (27 ± 4 years, 55.8 ± 10.4 mL/kg/min^−1^, *n* = 15) matched time (30 min) and increases in *T*_c_ (+1.4°C) across HWI (42°C), treadmill running and high‐intensity interval EXE on the 30‐min responses of FEM and cerebral shear rate. The results demonstrated that the three protocols elicited similar responses in FEM blood flow and caused minimal changes in the extracranial arteries of the brain. The authors concluded that HWI is likely a therapeutic alternative for individuals unable to perform EXE, as it imposes a lower cardiac workload than EXE, while still inducing similar acute haemodynamic changes (e.g., postexercise hypotension, altered shear patterns, and increased core temperature). These data lend support for the application of passive heating in clinical populations as a targeted therapeutic intervention to increase peripheral vascular shear.

We observed some sex differences in response to the sessions. Females exhibited greater FEM velocity and shear rate responses, as well as higher HR, after HWI compared to males, despite similar increases in *T*_c_, thermal comfort and sensation, and similar responses in the CCA and BRA arteries. Using a similar protocol, Larson et al. (2021) studied young males (24 ± 4 years, 23 ± 1 kg/m^2^, *n* = 11) and females (24 ± 4 years, 22 ± 2 kg/m^2^, *n* = 10) after 60 min of HWI (with arms out of the water). The authors concluded that both sexes exhibited similar increases in CCA artery parameters, *T*_c_, HR and BP, but that females had larger increases in BRA shear rate and velocity than males. This was attributed to the smaller diameter of conduit arteries in women and to the inverse relationship between shear stress and blood vessel diameter, which may promote a greater increase in shear stress with passive heating in women than in men. It is important to note that the study by Larson et al. (2021) did not assess FEM artery responses, limiting the ability to draw direct comparisons with our findings. In addition, in our study, females (+141 ± 51 s^−1^) had a higher increase in FEM antegrade shear than males (+86 ± 38 s^−1^) after HWI and compared to EXE session with no differences in retrograde shear (♀17 ± 35 vs. ♂25 ± 18 s^−1^, P = 0.531). This shift toward a more antegrade and less retrograde shear stress is known to be a direct mechanism responsible for the beneficial effects on the vasculature, improving endothelial function and facilitating blood vessel remodelling (Casey et al., 2016; Tuttle et al., 2001).

We observed that females demonstrated a larger rise in HR compared to males following HWI (48 ± 17 bpm vs. 27 ± 12 bpm). This finding aligns with the results of Atkins et al. (2024), where 12 older females exhibited a HR increase of 32 ± 9 bpm, which was higher than that of older males (18 ± 8 bpm), younger males (18 ± 6 bpm), and younger females (18 ± 12 bpm). These results suggest a potential interaction between age and sex. The decline in sex hormones during menopause is known to influence both sympathetic tone and thermoregulation (Keir et al., 2020). The postmenopausal females in our study may experience elevated sympathetic activity compared to their male counterparts, which may, in turn, lead to more reliance on blood flow to dissipate heat, necessitating higher cardiac output to maintain blood pressure. These factors may partially explain why females exhibited both higher HR and increased FEM blood flow velocity in our study, despite having *T*_c_ responses similar to those of males. Recognising the traditional under‐representation of female participants in physiology experiments, further research is required to enhance our understanding of the effects of EXE, heat exposure, and responses in pre‐ and post‐menopausal women (Hutchins et al., 2021).

Our study has several limitations. The 3‐min interval before arterial measurements may have affected results, potentially leading to an underestimation of the EXE effects on flow. However, the sessions were matched for the timing of all measurements, and we matched participants' posture during vascular assessments to ensure accurate and valid comparisons in a well‐controlled setting; it would be impossible to scan the FEM arteries while participants were in the water.

Regarding leg positioning *during* the interventions, postural differences may have contributed to the differences we observed between HWI and EXE. However, a previous study (Padilla et al., 2009) indicates that the passive effects of leg bending are relatively small after longer sessions than ours (3 h), particularly compared with the effects we observed.  In addition, if leg extension was associated with higher flow and shear through the femoral artery during HWI, we may have expected larger femoral diameters at the end of HWI versus EXE. This was not the case (see Figure 3b top). We acknowledge that immersing both arms in water did not allow us to distinguish local from systemic effects in response to the HWI session. However, this study was not designed to analyse isolated effects of heat and exercise stimulus but focused on maximising the thermoregulatory stimulus of the hot water. But our group have previously demonstrated (Carter et al., 2014b) that repeated episodic exposure to leg heating (8 weeks; 3 times per week) resulted in brachial artery vascular adaptation, which was dependent upon increases in shear stress. Another limitation was that in our sample five participants diagnosed with hypertension were under medication. This may affect their results. Our sample, therefore, reflects a real‐world population with a variety of comorbidities associated with overweight and obesity, which is critical for the generalizability of our findings. Our study results are also limited by the population we recruited; future studies may assess the effects of whole‐body heating in older participants with more compromised cardiovascular function, such as those with heart failure or diabetes (Green et al., 2006).

### 4.1 Conclusion

The present study demonstrated that HWI resulted in greater changes in BRA and FEM velocity and shear rate compared to cycle ergometer EXE of a similar duration. In addition, despite similar increases in *T*_c_, female participants had higher HR, FEM velocity and shear rate responses than males, demonstrating a higher antegrade shear response to 30 min of HWI. These findings highlight whole‐body heat therapy as a feasible, low‐intensity strategy to enhance vascular function and shear‐mediated endothelial stimuli in older, overweight adults with limited EXE tolerance.

## AUTHOR CONTRIBUTIONS

Juliene G. Costa: conceptualisation of the work; acquisition, analysis and interpretation of data for the work, writing—original draft. Joao Carlos Locatelli and Kristanti W. Wigati: conceptualisation of the work; acquisition, methodology, analysis and interpretation of data for the work; data curation; revised the manuscript critically for important intellectual content. Andrew Haynes and Louise H. Naylor: analysis and interpretation of data for the work; data curation; revised the manuscript critically for important intellectual content. Daniel J. Green: conceptualisation, supervision, data analysis, analysis and interpretation of data for the work and revised the manuscript critically for important intellectual content. All authors approved the final version of the manuscript; agreed to be accountable for all aspects of the work in ensuring that questions related to the accuracy or integrity of any part of the work are appropriately investigated and resolved; and all persons designated as authors qualify for authorship, and all those who qualify for authorship are listed.

## CONFLICT OF INTEREST

The authors reported no conflict of interest. The results of the study are presented clearly, honestly, and without fabrication, falsification or inappropriate data manipulation. The results of the present study do not constitute endorsement by the American College of Sports Medicine.

## GENERATIVE AI STATEMENT

The authors confirm that no artificial intelligence tools, including large language models (LLMs), were used in the drafting or revision of this manuscript. All content was conceived, written and approved solely by the authors.

## Supporting information



Supplementary Table 1. Baseline measurements for the experimental sessions.

## Data Availability

The source data are available to verified researchers upon request by contacting the corresponding author.
