# Supplementary material for: Sex differences in macrovascular responsiveness: Acute effects of hot water immersion versus aerobic exercise in overweight older adults
Source: Exp Physiol. 2026 Sep 25:10.1113/EP093959. Online ahead of print. doi: 10.1113/EP093959 (PMC13614612; doi:10.1113/EP093959)
Supplement: Supplementary file 1 [file EPH-9999-0-s001.docx]

| **Supplementary Table 1.** Baseline measurements for the experimental sessions | | | | | | | | | |  |
| --- | --- | --- | --- | --- | --- | --- | --- | --- | --- | --- |
|  | **Exercise - Baseline** | | |  | **HWI - Baseline** | | |  |  |  |
|  | All group (n=25) | Female (n=15) | Male (n=10) | F vs M  p-value | All group (n=25) | Female (n=15) | Male (n=10) | F vs M p-value | EXE vs HWI p-value |  |
| Tc (°C) | 37.0 ± 0.3 | 37.0 ± 0.3 | 37.0 ± 0.4 | 0.756 | 37.1 ± 0.3 | 37.2 ± 0.3 | 37.1 ± 0.2 | 0.886 | 0.101 |  |
| SBP (mmHg) | 128 ± 16 | 122 ± 14 | 130 ± 16 | 0.208 | 129 ± 13 | 129 ± 12 | 129 ± 15 | 0.976 | 0.246 |  |
| DBP (mmHg) | 74 ± 10 | 70 ± 9 | 80 ± 9 | 0.012 | 73 ± 10 | 70 ± 9 | 78 ± 11 | 0.070 | 0.981 |  |
| HR (beats/min) | 67 ± 10 | 69 ± 9 | 64 ± 11 | 0.302 | 68 ± 14 | 65 ± 10 | 71 ± 19 | 0.357 | 0.712 |  |
| Brachial |  |  |  |  |  |  |  |  |  |  |
| Diameter (mm) | 3.63 ± 0.64 | 3.33 ± 0.31 | 4.04 ± 0.78 | 0.005 | 3.63 ± 0.62 | 3.31 ± 0.44 | 4.08 ± 0.56 | 0.001 | 0.980 |  |
| Velocity (cm/s) | 15.6 ± 7.5 | 18.5 ± 8.1 | 11.5 ± 4.2 | 0.022 | 15.2 ± 9.8 | 17.9 ± 11.1 | 11.4 ± 6.4 | 0.109 | 0.847 |  |
| Flow (mL/min) | 95 ± 47 | 99 ± 52 | 89 ± 41 | 0.606 | 94 ± 62 | 95 ± 64 | 91 ± 61 | 0.890 | 0.878 |  |
| Shear rate (1/s) | 179 ± 94 | 221 ± 96 | 119 ± 51 | 0.006 | 174 ± 118 | 217 ± 131 | 113 ± 60 | 0.028 | 0.826 |  |
| Femoral |  |  |  |  |  |  |  |  |  |  |
| Diameter (mm) | 6.61 ± 1.25 | 6.15 ± 1.09 | 7.30 ± 1.19 | 0.021 | 6.78 ± 1.21 | 6.29 ± 1.12 | 7.29 ± 1.12 | 0.083 | 0.949 |  |
| Velocity (cm/s) | 10.5 ± 7.43 | 12.4 ± 8.5 | 7.7 ± 4.6 | 0.123 | 9.4 ± 3.5 | 9.9 ± 3.3 | 8.6 ± 3.8 | 0.409 | 0.390 |  |
| Flow (mL/min) | 219 ± 167 | 248 ± 206 | 176 ± 72 | 0.229 | 202 ± 82 | 193 ± 74 | 214 ± 95 | 0.561 | 0.365 |  |
| Shear rate (1/s) | 65 ± 44 | 78 ± 48 | 45 ± 31 | 0.065 | 59 ± 31 | 65 ± 33 | 50 ± 27 | 0.240 | 0.547 |  |
| Carotid |  |  |  |  |  |  |  |  |  |  |
| Diameter (mm) | 6.87 ± 0.70 | 6.67 ± 0.76 | 7.15 ± 0.52 | 0.098 | 6.81 ± 0.91 | 6.44 ± 0.80 | 7.28 ± 0.86 | 0.024 | 0.937 |  |
| Velocity (cm/s) | 29.6 ± 7.7 | 31.1 ± 8.5 | 27.6 ± 6.4 | 0.284 | 32.2 ± 9.7 | 33.9 ± 8.7 | 29.9 ± 10.8 | 0.334 | 0.125 |  |
| Flow (mL/min) | 651 ± 136 | 647 ± 153 | 6587 ± 116 | 0.852 | 691 ± 208 | 663 ± 196 | 728 ± 227 | 0.471 | 0.246 |  |
| Shear rate (1/s) | 176 ± 58 | 191 ± 66 | 156 ± 42 | 0.158 | 195 ± 72 | 216 ± 72 | 168 ± 66 | 0.117 | 0.100 |  |
| Values are presented as mean ± standard deviation. EXE: exercise; HWI: hot water immersion; Tc: core temperature; SBP: systolic blood pressure; DBP: diastolic blood pressure; HR: heart rate. EXE vs HWI Paired T-test; F vs M Unpaired T-test; significance was set at p <0.05. | | | | | | | | | |  |
|  |  |  |  |  |  |  |  |  |  |  |
|  |  |  |  |  |  |  |  |  |  |  |
